# Regulation of T Cell Trafficking by Enzymatic Synthesis of O-Glycans

**DOI:** 10.3389/fimmu.2017.00600

**Published:** 2017-05-24

**Authors:** Samuel J. Hobbs, Jeffrey C. Nolz

**Affiliations:** ^1^Department of Molecular Microbiology and Immunology, Oregon Health and Science University, Portland, OR, United States; ^2^Department of Cell, Developmental and Cancer Biology, Oregon Health and Science University, Portland, OR, United States; ^3^Department of Radiation Medicine, Oregon Health and Science University, Portland, OR, United States

**Keywords:** T cell trafficking, O-glycans, selectins, high endothelial venules, T cell memory, sialyl Lewis X, PSGL-1, inflammation

## Abstract

Selectins constitute a family of oligosaccharide binding proteins that play critical roles in regulating the trafficking of leukocytes. In T cells, L-selectin (CD62L) controls the capacity for naive and memory T cells to actively survey peripheral lymph nodes, whereas P- and E-selectin capture activated T cells on inflamed vascular endothelium to initiate extravasation into non-lymphoid tissues. The capacity for T cells to interact with all of these selectins is dependent on the enzymatic synthesis of complex O-glycans, and thus, this protein modification plays an indispensable role in regulating the distribution and homing of both naive and previously activated T cells *in vivo*. In contrast to neutrophils, O-glycan synthesis is highly dynamic in T cell populations and is largely controlled by extracellular stimuli such as antigen recognition or signaling though cytokine receptors. Herein, we review the basic principles of enzymatic synthesis of complex O-glycans, discuss tools and reagents for studying this type of protein modification and highlight our current understanding of how O-glycan synthesis is regulated and subsequently impacts the trafficking potential of diverse T cell populations.

## Introduction

Protein glycosylation occurs through the collective action of glycosyltransferases and glycosidases that are active in the lumen of the endoplasmic reticulum or the golgi complex. This protein modification has been shown to impact a variety of immunological processes, including lymphocyte development, trafficking, apoptosis, antigen recognition, and cellular response to cytokines (1). The number of possible, unique glycan modifications is vast and largely not understood, but can be generalized into two broad categories based on the amino acid from which the glycan modification originates. N-linked glycosylation is the most common form of glycosylation, is initiated from an asparagine residue through a β-N glycosidic bond, and is always found in the context of an NXT/S motif. In contrast, O-linked glycans do not require a bona fide consensus motif and are covalently attached to the oxygen atom from the hydroxyl group of serine or threonine amino acids. Among the many diverse functions for O-glycans in regulating many aspects of T cell biology, the most characterized role for these post-translational modifications is for capturing circulating T cells on vascular endothelium and allowing them to infiltrate both lymphoid and non-lymphoid tissues (2, 3).

## Selectins and Selectin Ligands

Selectins are a family of C-type lectin domain containing proteins that bind specific carbohydrate structures in a calcium-dependent manner (4). These proteins are responsible for the initial capture (i.e., “rolling”) of leukocytes from the circulation before extravasation across the vascular endothelium can occur. Each selectin is named according to its expression pattern. E-selectin (CD62E) is expressed on endothelial cells and P-selectin (CD62P) is stored in α-granules in platelets (as well as Weibel-Palade bodies in endothelial cells). Following infection or tissue injury, both P- and E-selectin are expressed by the vascular endothelium in response to inflammatory cytokines such as IL-1 and TNFα (5). In contrast, L-selectin (CD62L) is constitutively expressed on many leukocytes. All of the selectins share a common general structure that consists of an amino-terminal lectin domain that is responsible for ligand binding, an epidermal growth factor-like domain, a variable number of consensus repeats, a transmembrane domain, and a cytoplasmic domain (6).

Selectin ligands can be divided into two functional categories—those that have a role in steady-state lymphocyte homing to secondary lymphoid organs and those that have a role in the capture of leukocytes in response to inflammation or tissue injury. Lymphocyte homing to lymph nodes is primarily mediated by CD62L ligands that are constitutively expressed by endothelial cells in high endothelial venules (HEVs), while leukocyte homing into non-lymphoid tissue is mediated largely by P- and E-selectin ligands (7, 8). Several proteins have been demonstrated to bind CD62L and mediate steady-state lymphocyte homing into lymph nodes including GlyCAM-1, CD34, spg200, podocalyxin, and endomucin (3), and, based on the O-glycans they are decorated with, are collectively referred to as peripheral node addressins (PNAd). P-selectin glycoprotein ligand-1 (PSGL-1) is the primary ligand for P-selectin and can also bind to both L- and E-selectin (9). Recently, the T cell immunoglobulin and mucin domain glycoprotein 1 has also been shown to bind P-selectin, when expressed by CD4^+^ T cells (10). Several other leukocyte-expressed proteins besides PSGL-1 are capable of binding E-selectin, including E-selectin ligand-1 (ESL-1), CD44, and CD43 (11). In all cases, the unmodified selectin ligand is unable to bind to selectins, rather this interaction is dependent on the presence of specific O-linked glycosylation modifications.

## Enzymatic Synthesis of O-Glycans

**Figure 1:**
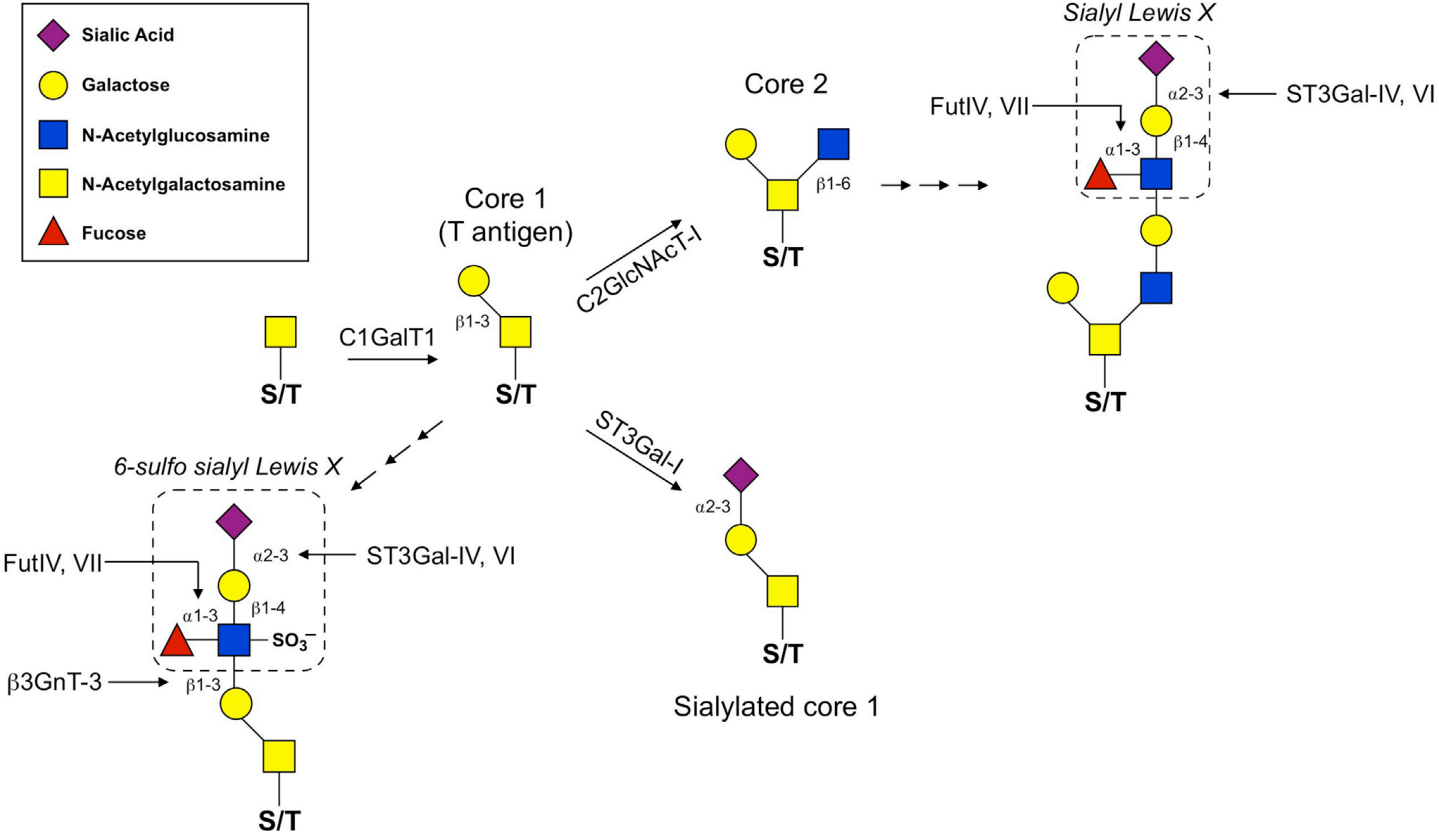
**O-glycan synthesis pathways used for the generation of selectin ligands**. O-glycan synthesis originates from the hydroxyl group of a serine or threonine (S/T) amino acid by the addition of *N*-acetylgalactosamine. C1GalT1 adds a β1,3-linked galactose to the initial O-GalNAc to generate the T antigen. From here, core 1 can be extended by β3GnT-3 and the formation of the 6-sulfo sLe^x^ generates the ligands that react with the MECA-79 antibody used to identify high endothelial venules. C2GlcNAcT-I and ST3Gal-I compete for the T antigen as a substrate. ST3Gal-I activity catalyzes the addition of an α2,3-linked sialic acid to the β1,3-linked galactose, which prevents core 2 extension. Alternatively, C2GlcNAcT-I adds a β1,6-linked N-acetylglucosamine that can then be extended with poly-N-lactosamine residues and ultimately decorated with a sLe^x^ motif. For details of glycosyltransferase nomenclature, see also Table 1.

Synthesis of O-glycans is initiated by the attachment of *N*-acetylgalactosamine (GalNAc) to the oxygen molecule of the hydroxyl group present on serine or threonine amino acids. From there, O-glycans can contain a wide variety of modifications, but the most common are those which contain one of four core structures branching from the initial GalNAc (Figure 1; Table 1). Of these four core structures, core 1 and core 2 have been implicated in a variety of immunological processes, including T cell contraction by apoptosis, antibody function, antigen receptor activation, and cell adhesion and trafficking (12–14). On the other hand, the formation of core 3 and core 4 structures is far less prevalent and no clear role for these O-glycan types have been shown to contribute significantly to the regulation of innate or adaptive immunity.

**Table 1:** **Mice with gene-targeted deficiencies for enzymes involved in the synthesis of O-glycans**.

Gene	Full enzyme name	Abbreviated name(s)	Function	Reference
*Gcnt1*^a^	Core 2 β1-6 *N*-acetylglucosaminyltransferase-I	C2GlcNAcT-I; C2GnT-I	Initial β1-6 GlcNAc for core 2 O-glycans	Ellies et al. (15)
*Fut7*	α1-3 fucosyltransferase VII	FutVII; FucTVII	Fucosyltransferase for Lewis X synthesis (Major)	Maly et al. (16)
*Fut4*	α1-3 fucosyltransferase IV	FutIV; FucTIV	Fucosyltransferase for Lewis X synthesis (Minor)	Homeister et al. (17)
*St3gal4*	ST3 β-galactoside α2-3 sialyltransferase IV	ST3Gal-IV	Sialylation of Lewis X (Major)	Ellies et al. (18)
*St3gal6*	ST3 β-galactoside α2-3 sialyltransferase VI	ST3Gal-VI	Sialylation of Lewis X (Minor)	Yang et al. (19)
*B3Gnt3*	β1-3 *N*-acetylglucosaminyltransferase-3	β3GnT-3	Extension of Core 1 O-glycans	Yeh et al. (20)
*St3Gal1*^a^	ST3 β-galactoside α2-3 sialyltransferase-I	ST3Gal-I	Core 1 sialylation	Priatel et al. (21)

*^a^Mice with conditional potential available*.

The diversity of O-glycan structures is achieved through the activity of glycosyltransferase enzymes that catalyze the addition of monosaccharides in a sequential, stepwise fashion (Figure 1). The expression of these enzymes is generally regulated at the transcriptional level and is often cell-type and tissue specific (22). To generate a core 1 O-glycan, core 1 β1-3 galactosyltransferase (C1GalT1) adds a galactose to the initial GalNAc in a β1-3 linkage. The resulting unmodified core 1 structure is also referred to as the T antigen and is rarely left unmodified. The potential modifications that can be added to the T antigen are extension, capping, or it can be used as a substrate for synthesis of core 2 O-glycans. Capping of core 1 with α2,3-linked sialic acid requires *ST3Gal1* in T cells and this modification inhibits the synthesis of core 2 O-glycans (23). Mechanistically, this is because core 2 β1-6 *N*-acetylglucosaminyltransferase (C2GlcNAcT), the enzyme responsible for core 2 synthesis, requires unmodified core 1 as a substrate and catalyzes the addition of an *N*-acetylglucosamine (GlcNAc) to unmodified core 1 via a β1–6 linkage. There are three enzymes in the C2GlcNAcT family, C2GlcNAcT-I, -II, and -III, which are transcribed from the genes *Gcnt1, Gcnt3*, and *Gcnt4*, respectively. (Note: *Gcnt2* is an *N*-acetylglucosaminyltransferase but does not exhibit core 2 activity.)

Extension of both core 1 and core 2 is catalyzed by *N*-acetylglucosaminyltransferases and galactosyltransferases, which add repeated units of *N*-acetyllactosamine to the galactose of core 1 or the GlcNAc of core 2. Poly-*N*-acetyllactosamine repeats are more commonly found on core 2 O-glycans and can serve as acceptor sites for further modifications. One such modification that is critical for selectin ligand formation is the addition of the tetrasaccharide Lewis X (Le^x^) to extended core 2 O-glycans. The most basic form of the Le^x^ antigen consists of a fucose bound to the *N*-acetyllatosamine of the Le^x^ by an α1-3 linkage. There are a diverse set of more complex Lewis antigens utilized throughout biology, but within the scope of those that impact selectin binding, two forms are the most relevant. First, P- and E-selectin ligands have a sialyl Lewis X (sLe^x^) which contains a sialic acid bound to the terminal galactose of the Lewis antigen in an α2-3 linkage, catalyzed by the α2-3 sialyltransferases (ST3Gal) family of enzymes (18). The other form is a sulfated version (6-sulfo sLe^x^), which contains a sulfate attached to C6 of the GlcNAc of the Lewis antigen and is present on PNAd (CD62L ligands). Thus, terminal sialic acids and fucoses (as well as GlcNAc sulfation) that are attached to the poly-*N*-acetyllactosamine repeats of complex O-glycans are critical for generating the functional binding motif for all selectins.

## Tools for Interrogating O-Linked Glycosylation

The ability to identify specific glycan structures is paramount to understanding and investigating the role of glycans in biology. To this end, lectins and monoclonal antibodies can be used in glycan analysis, at both the cellular and protein level. Lectins are a class of proteins (typically isolated from plants) that bind general features of carbohydrates (e.g., specific linkages between two monosaccharides), but typically cannot identify highly specific or complex glycan determinants. The identification of a specific complex, protein-associated glycan requires a monoclonal antibody (mAb), but these reagents are difficult to generate and are less available than lectins (24). Finally, the use of glycosidases can be used to cleave specific glycosidic bonds and allow one to assess functional roles for saccharides. For example, the presence of sialic acid on sLe^x^ is critical for selectin binding, as rolling of leukocytes and the generation of P- and E-selectin ligands is greatly diminished after treatment with neuraminidase (25). Ultimately, however, the generation of gene-deficient animals is required to designate meaningful biological functions to individual glycosyltransferases that regulate the trafficking of T cells during both homeostasis or infections/inflammation (Table 1).

Fluorescently labeled lectins and antibodies can be used in flow cytometry or imaging to determine the expression and distribution of glycan patterns in cells or in tissue, but their utility extends beyond these applications. Lectins covalently linked to agarose can also be used in affinity purification of a protein or cell of interest. As lectins are typically isolated from plant seeds, they are generally cheaper than mAbs and are therefore well suited for purification applications. The lectins phytohemagluttinin from *Phaseolus vulgaris* and Concanavalin A (ConA) bind to complex N-glycans and can be used as potent mitogens for expanding and/or activating T cells *in vitro*.

**Figure 2:**
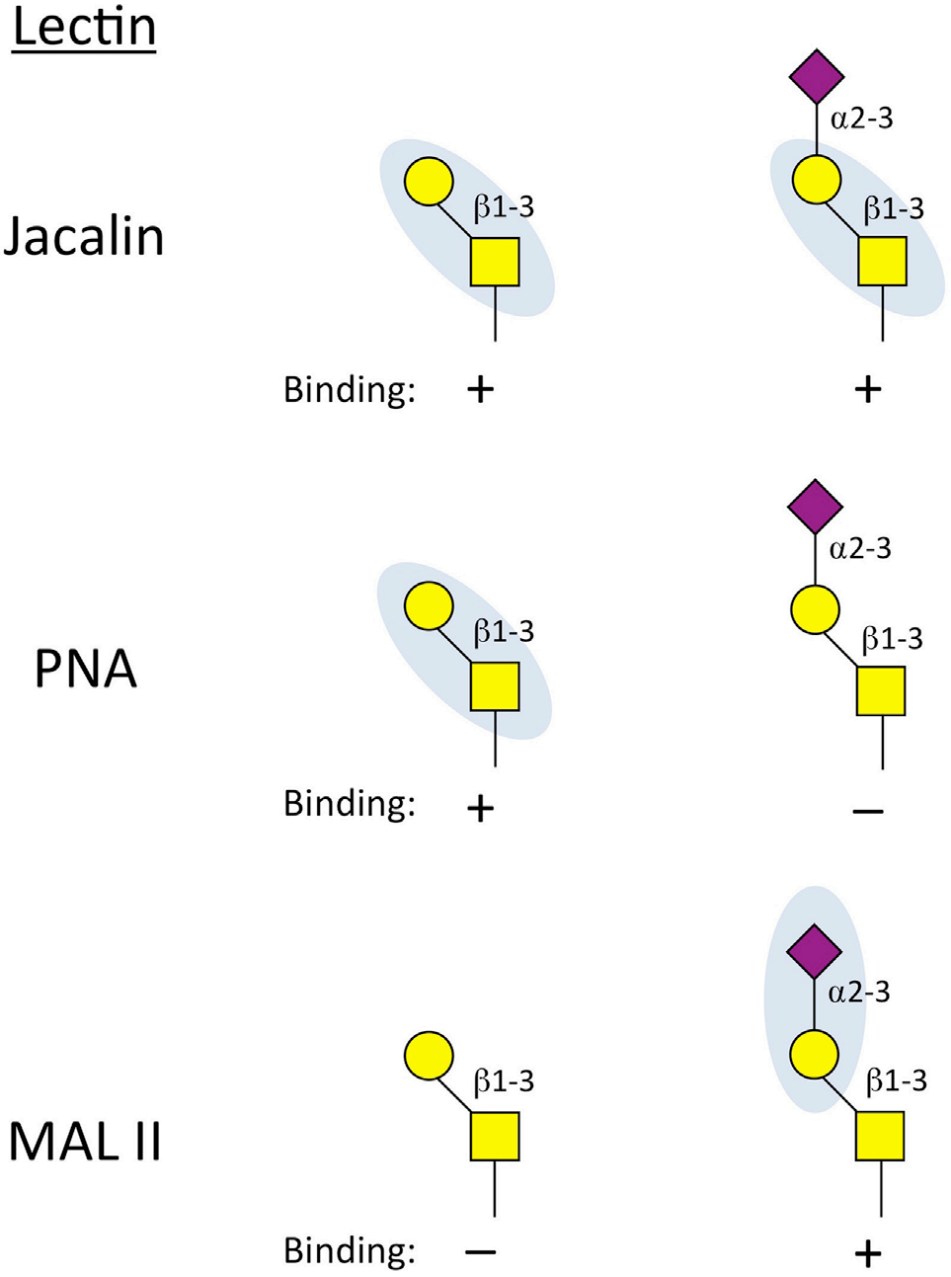
**Binding properties of lectins used to interrogate core 1 O-glycan status**. Jacalin can bind the unmodified core 1 base regardless of whether it is sialylated. Peanut agglutinin (PNA) will only bind core 1 O-glycans when the α2,3-sialic acid is not present. *Maackia amurensis* lectin II (MAL II) reacts to the α2-3 sialic acid linked to the β1,3-galactose of core 1 O-glycans. Together, this panel of lectins can determine if core 1 contains the sialic acid cap (Jacalin^+^, MAL II^+^) and whether it is possible that core 2 is present (core 2 requires unmodified core 1 as a substrate and therefore can only be present on PNA^+^ and MAL II^–^cells).

There are a large number of commercially available lectins and their binding specificities cover a wide range of glycan determinants. For example, *Erythrinia cristagalli* lectin binds to Galβ1-R (terminal galactose) structures, whereas tomato (LEL) and potato (STL) lectins bind to poly-*N*-acetyllactosamine repeats, generated on either N- or O-glycans. *Aleuria aurantia* lectin binds terminal fucoses such as the α1,3-linkage found in sLe^x^, but reportedly has the highest affinity for α1,6-linked fucose (26), a feature of many complex N-glycans. Although they are typically specific for only short or even individual saccharide motifs, the wide range of determinants covered by lectins allows them to be used in combination to reveal specific glycan structures. For example, a combination of Jacalin, peanut agglutinin (PNA), and *Maackia amurensis* lectin II (MAL II) can be used to determine the sialylation state of core 1 O-glycans on a cell surface or protein. Jacalin will bind the T antigen whether or not is sialylated, while PNA will only bind the unsialylated T antigen (Figure 2). Conversely, MAL II is specific for the α2,3-linked sialic acid attached to the core 1 β1,3-galactose (27). Thus, a loss of Mal II binding, a gain in PNA binding and no change in Jacalin binding would collectively indicate an increase of unsialylated core 1 O-glycans.

The development of monoclonal antibodies that are able to recognize specific glycan motifs on individual proteins has not been rigorously pursued. However, several mAb specific for each of the selectins (both for human and mice) have been generated that can be used to analyze expression and to functionally inhibit receptor–ligand interactions *in vitro* and *in vivo* (Table 2). In addition to antibodies against selectins, there are some antibodies that recognize glycosylation patterns on proteins. The ligand for the HECA-452 mAb is “cutaneous lymphocyte antigen” (CLA), which is often used in human samples to identify T cells that can bind to E-selectin and have “skin homing potential” (28, 29). MECA-79 is a mAb that reacts to 6-sulfo Le^x^ on core 1 O-glycans and is used to identify HEVs (or HEV-like structures) and this antibody can sufficiently block naive T cell homing to secondary lymphoid organs *in vivo* (30). Finally, the mAb 1B11 binds mouse CD43 only when modified with core 2 O-glycans (31). In fact, in T cells, 1B11 reactivity has been shown to require *Gcnt1*, the enzyme responsible for the initiation of core 2 O-glycan synthesis (32).

**Table 2:** **Examples of monoclonal antibodies for studying O-glycosylation and selectin-mediated trafficking in mice**.

Target	Clone	Reference
L-selectin (CD62L)	MEL-14	Gallatin et al. (33)
P-Selectin (CD62P)	RB40.34	Bosse and Vestweber (34)
E-Selectin (CD62E)	10E9.6	Ramos et al. (35)
E-Selectin (CD62E)	9A9	Norton et al. (36)
PSGL-1	4RA10	Frenette et al. (37)
PSGL-1	2PH1	Borges et al. (38)
O-Glycosylated CD43	1B11^a^	Jones et al. (39)
PNAd (sulfated core 1 O-glycans)	MECA-79	Streeter et al. (30)

*^a^1B11 will also bind to an unsialylated form of CD45*.

## Dynamic O-Glycan Synthesis Regulates the Trafficking of T Cells

In contrast to cells of the innate immune system (e.g., neutrophils and monocytes), which often constitutively express the collection of enzymes that generate selectin ligands on their cell surface, the synthesis of core 2 O-glycans and the expression of CD62L in T cells is highly dynamic. As mentioned previously, the regulation of glycosyltransferase functional activity is believed to occur largely at the transcriptional level, where the enhancement (or inhibition) of enzyme expression generally controls the surface glycan landscape of a cell. Thus, the signaling mechanisms that ultimately impact the transcriptional and/or epigenetic regulation of glycosyltransferase expression in both T cells and endothelial cell populations is critical for the overall understanding of how specific T cell populations are able to traffic into both lymphoid and non-lymphoid tissues (40).

## Recruitment of T Cell Precursors Into the Thymus

Before they can populate the periphery, hematopoietically derived T cell precursors exit the bone marrow and must home to the thymus to undergo positive and negative selection to mature into either a CD8^+^ or CD4^+^ T cell. Interestingly, thymic endothelial cells express P-selectin and the ability for T cell precursors to generate P-selectin ligands is critical for allowing these cells to home to the thymus (41). Parabiotic experiments that combined the circulation of either *Gcnt1*, PSGL-1, or P-selectin deficient mice with wild-type (WT) mice revealed that WT T cell precursors were able to populate *Gcnt1-* and PSGL-1-deficient thymuses, but not thymuses that lacked P-selectin. Conversely, P-selectin deficient T cell precursors were able to populate thymuses independent of thymically expressed *Gcnt1* and PSGL-1. Thus, this eloquent study demonstrated that *Gcnt1*-dependent modification of PSGL-1 on T cell precursors generates a ligand that can bind thymic endothelial P-selectin that is required for their homing to the thymus.

## Trafficking of T Cells Into Lymph Nodes

Following their development and exit from the thymus, antigen-naive T cells enter the periphery where they continually survey the spleen and secondary lymphoid organs for an encounter with cognate antigen. Naive T cells express high levels of CD62L and, in fact, are typically defined as being CD44^Lo^/CD62L^Hi^ in mice and CD45RA^+^/CD62L^Hi^ in humans. Once a naive T cell is activated by antigen, co-stimulation, and inflammatory cytokines, a rapid proliferative burst gives rise to clonally expanded effector T cells that gain effector functions (e.g., cytotoxicity, cytokine production) and lose expression of CD62L by suppressed transcriptional activity and protease-mediated cleavage (42, 43). Most effector T cells have a limited life span and are eliminated by apoptosis during the contraction phase of the response, but 5–10% of these cells survive to become the long-lived memory T cell population (44, 45).

Expression of CD62L, along with the chemokine receptor CCR7, categorizes memory T cells into either the central (T_CM_) or effector (T_EM_) memory subsets in both humans and mice (46, 47). T_CM_ express CD62L and actively survey lymph nodes, whereas T_EM_ do not express CD62L and their distribution is therefore limited to the circulation, spleen, and non-lymphoid tissues. This distribution of trafficking potential within the memory T cell compartment has been postulated to maximize the tissue surveillance of these protective T cells, so that both lymph nodes and peripheral tissues can be patrolled for invading pathogens. In fact, CD62L^−/−^ memory CD8^+^ T cells are unable to provide protective immunity against chronic viral infection in lymph nodes, but provide complete protection against *Listeria monocytogenes* infection of the spleen and liver (48). Thus, there is utility in using CD62L expression to identify T cells subsets and also demonstrates the functional importance of this gene in regulating the distribution of memory T cell populations *in vivo*.

Naive and T_CM_ T cells exit the circulation and enter lymph nodes by crossing specialized vascular endothelium known HEVs. PNAd are the functional ligands for CD62L expressed by the HEV and rely on the synthesis of sulfated core 1 O-glycans on proteins such as GlyCAM and CD34. The complete gene expression profile of HEV endothelial cells isolated from peripheral lymph nodes has now been reported (49). As expected, the cells express enzymes that stimulate O-glycan synthesis including *C1GalT1, Gcnt1, B3Gnt3, Fut7*, and several *B4GalT*s, all which could contribute to the biosynthesis of CD62L ligands. Interestingly, studies aimed to identify a requirement for individual glycosyltransferases in generating CD62L ligands for T cell trafficking across HEVs have been less clear. β3GnT-3 is required for extending core 1 O-glycans and HEVs in *B3Gnt3*^−/−^ mice lose reactivity to the MECA-79 antibody (20). Furthermore, HEVs from mice deficient in both *B3Gnt3* and *Gcnt1* lose essentially all extended O-glycans (both core 1 and core 2), but surprisingly, naive T cell trafficking into peripheral lymph nodes is reduced by only ~50% (50). However, because naive T cell trafficking into lymph nodes is CD62L-dependent, it was found that CD62L ligands could also be formed on complex N-glycans. In contrast, the α1,3-fucosyltransferases *Fut7*/*Fut4* and the *N*-acetylglucosamine-6-*O*-sulfotransferases *GlcNAc6ST-1/-2* are more essential for naive T cell homing into lymph nodes (16, 17, 51–53), thereby demonstrating that the formation of 6-sulfo sLe^x^ is critical, but can be synthesized on both O- and N-glycans. Overall, these findings suggest that there are several redundant glycosylation mechanisms that can ultimately recruit CD62L-expressing T cells into lymph nodes. However, the fact that the MECA-79 antibody is efficient at blocking T cell trafficking into lymph nodes (30) suggests that sulfated core 1 O-glycans are the primary ligands for CD62L, but in their absence, other glycan types decorated with sulfated sLe^x^ can facilitate the capture of naive T cells on HEVs.

Lymphatic linkage between peripheral tissues and draining lymph nodes is essential for maintaining HEVs and overall lymph node integrity (54–56). Thus, the vascular endothelial cells that comprise HEVs are not intrinsically programmed to express the adhesion molecules and glycosyltransferase enzymes that generate CD62L ligands, but require extrinsic cellular and/or microenvironmental factors. It has been known for several years that lymphotoxin-α and the lymphotoxin β receptor are critical for the development of mature, functional lymph nodes (57, 58). Recently, it has now been uncovered that dendritic cells are the cellular source of lymphotoxin for maintaining HEVs in peripheral lymph nodes and depletion of CD11c^+^ dendritic cells results in a loss of MECA-79 reactivity, decreased size and cellularity of peripheral lymph nodes, and impaired homing of naive T cells (59). Lymphotoxin α from CD11c^+^ dendritic cells that have migrated with the afferent lymph to the draining lymph node stimulate the expression of *Fut7, GlcNAc6ST-2*, and *GlyCAM* on HEVs, but interestingly, does not regulate the expression of other adhesion molecules such as *ICAM-1, CD31*, or *VE-cadherin*.

Because CD11c^+^ dendritic cells stimulate lymph node endothelial vessels to express sulfated sLe^x^ suggests that HEV-like structures could potentially form in other tissues besides lymph nodes depending on the local inflammatory context. In fact, HEV-like structures (as defined by reacting with MECA-79) that form in non-lymphoid tissues are often referred to as tertiary lymphoid tissue or mucosa-associated lymphoid tissue (60). Tertiary lymphoid structures have received considerable attention in recent years, particularly in the field of cancer immunology, where these structures may support tumor-specific T cell activation and their presence is generally predictive of favorable treatment outcome (61, 62). Thus, the synthesis of sulfated core 1 O-glycans can occur in tissues besides lymph nodes and could regulate the trafficking and potentially the activation of CD62L-expressing T cells during local inflammatory events in non-lymphoid tissue.

## T Cell Activation Stimulates Core 2 O-Glycan Synthesis

**Figure 3:**
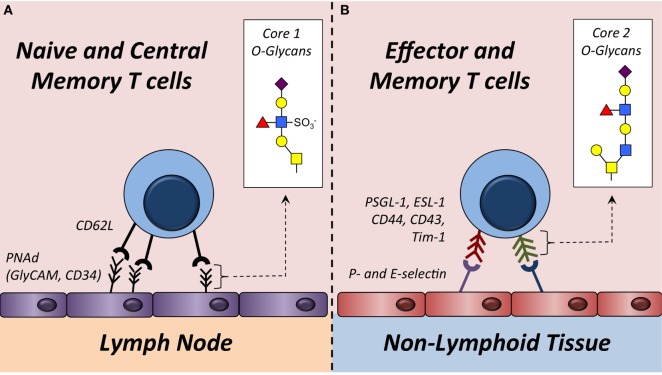
**Trafficking of T cells into lymph nodes or non-lymphoid tissue is regulated by O-glycan synthesis**. (**A**) Naive and some memory T cells express L-selectin (CD62L). This serves as the receptor for ligands (e.g., GlyCAM-1, CD34, etc.) expressing sulfated core 1 O-glycans in the high endothelial venules of lymph nodes. Sulfated core 1 O-glycans are collectively referred to as peripheral node addressins (PNAd). (**B**) Effector and some memory T cells synthesize core 2 O-glycans on surface proteins including PSGL-1 and CD43. These modified proteins function as P- and/or E-selectin ligands that facilitate capture of these T cells on activated vascular endothelium to initiate extravasation into non-lymphoid tissues. Enzymes required to synthesize core 1 and core 2 O-glycans are described in Figure 1 and Table 1.

Naive T cells cannot synthesize core 2 O-glycans or bind to P- and E-selectin, which essentially excludes them from entering non-lymphoid tissues. Following stimulation of the T cell receptor, both CD8^+^ and CD4^+^ T cells increase expression of *Gcnt1, Fut7*, and likely additional enzymes that facilitate core 2 O-glycan synthesis (Figure 1). This post-translational modification transforms surface proteins such as PSGL-1 and CD43 into P- and E-selectin ligands to direct extravasation across activated vascular endothelium and into non-lymphoid tissues. Although T cell migration is thought to be largely dictated by O-glycan modifications on selectin ligands, in neutrophils, N-linked glycosylation of both CD44 and ESL-1 has been shown to contribute to E-selectin ligand formation (63, 64). Even though the formation of P- and E-selectin ligands on T cells may not be completely dependent on O-glycan synthesis, the cellular source of the glycosylation for T cells to traffic into non-lymphoid tissue is the opposite of that needed by naive and T_CM_ T cells to home into lymph nodes. Specifically, naive and T_CM_ T cells require CD62L ligands to be synthesized on HEVs, whereas synthesis of complex core 2 O-linked glycans on both effector and memory T cells dictates the capacity for these cells to be captured by P- and/or E-selectin to initiate the extravasation process and ultimately infiltrate non-lymphoid tissues (Figure 3).

Following activation, most effector CD8^+^ T cells express *Gcnt1*, core 2 O-glycans, and generate P- and E-selectin ligands. A number of *in vitro* studies of CD8^+^ T cell activation have shown that cytokines such as IL-2 and IL-12 can stimulate further P-selectin binding activity (65, 66). However, in a model of autoimmunity, where TCR-transgenic CD8^+^ T cells from female mice specific for the male HY-antigen are transferred into male mice, IL-2, -12, and -15 were all dispensable for the formation of P-selectin ligands (67), arguing that antigen recognition may be sufficient to stimulate core 2 O-glycan synthesis in CD8^+^ T cells. Whether these cytokines contribute to P-selectin ligand formation on antigen-specific CD8^+^ T cells responding to active viral or bacterial infection has not been examined in detail. Core 2 O-glycan synthesis is regulated primarily by C2GlcNAcT-I in CD8^+^ T cells, but C2GlcNAcT*-*III may also contribute (68). Following activation, effector CD8^+^ T cells increase reactivity to PNA (69), demonstrating that the process of CD8^+^ T cell activation results in decreased capping of the α2,3-linked sialic acid on core 1 O-glycans (Figure 2). Because ST3Gal-I and C2GlcNAcT-I compete for the same core 1 O-glycan substrate (21, 70), this change in core 1 O-glycosylation status could be because of increased C2GlcNAcT-I expression, suppressed ST3Gal-I expression, or perhaps both. Notably, most *St3gal1*^−/−^ naive CD8^+^ T cells do not survive in the periphery (21), demonstrating that O-linked glycosylation plays important roles in T cell biology beyond regulating their trafficking potentials.

In contrast to cytotoxic CD8^+^ T cells, CD4^+^ T cells can be polarized *in vitro* and *in vivo* to become T-helper (Th)1, Th2, Th17 or FoxP3^+^ regulatory T cells (Treg). The collective agreement from a number of studies suggest that Th1 CD4^+^ T cells differentiated with IL-12 express P-selectin ligands and, to a lesser extent, E-selectin ligands, whereas Th2 CD4^+^ T cells generated with IL-4 do not. Mechanistically, the transcription factors T-bet and STAT4 (which is activated by IL-12) drive the expression of *Gcnt1* and stimulates the synthesis of P-selectin ligands (71). Interestingly, it has been reported that E-selectin ligand formation on Th1 CD4^+^ T cells does not require *Gcnt1*, suggesting that CD4^+^ T cells may generate E-selectin ligands independent of core 2 O-glycan synthesis (72). Besides IL-12, a number of other inflammatory cytokines may also influence the generation of P- and E-selectin ligands on CD4^+^ T cells. *In vitro*, IL-18, IL-27, IL-9, IL-25, and TGF-β1 all stimulate expression of *Gcnt1* and *Fut7* in a p38-dependent manner (73). Importantly, activation of the MAPK p38 by these cytokines requires concurrent TCR stimulation, further supporting the concept that an antigen encounter is critical to allow a previously naive T cell to begin synthesizing core 2 O-glycans.

In recent years, a number of additional CD4^+^ T cell lineages have been characterized, which has expanded our understanding of helper T cell biology beyond the classical Th1/Th2 differentiation paradigm. CD4^+^ T cells activated in the presence of TGF-β and IL-6 become IL-17-producing Th17 cells, which are critical for mucosal host defense, but have also been implicated in causing aberrant immunopathology in the skin and gut (74). A recent study showed that Th17 CD4^+^ T cells synthesize E-selectin ligands better than Th1 CD4^+^ T cells (75), which is consistent with the observation that Th17 cells are often found in non-lymphoid tissue. However, the fundamental glycobiology (e.g., N-linked vs. O-linked, core 1 vs. core 2, etc.) facilitating E-selectin ligand synthesis of TGF-β and IL-6 differentiated Th17 CD4^+^ T cells has not been defined. The regulation of core 2 O-glycan synthesis in CD4^+^ Tregs also remains largely unexplored, although it has recently been reported that Tregs bearing sLe^x^ are the most suppressive Treg “subset” found in humans (76). This interesting finding suggests that the tissue homing potential may be directly related to Treg function and that the capacity for these cells to traffic into non-lymphoid tissue may be necessary for their ability to suppress ongoing immune responses. Overall, these studies conclude that TCR-mediated activation is essential for allowing previously naive T cells to begin synthesizing core 2 O-glycans, but that a variety of cytokines and the process of Th-differentiation is critical for shaping the transcriptional activity of specific glycosyltransferase genes that ultimately control P- and E-selectin ligand formation.

## Trafficking of T Cells Into Non-Lymphoid Tissues

P- and E-selectin ligands that are synthesized on recently activated T cells have been shown to regulate their trafficking into a variety of non-lymphoid tissues during inflammatory challenges. Infiltration of the skin, in particular, relies heavily on the expression of P- and E-selectin ligands (77–79), but expression of these ligands has also been implicated in controlling T cell trafficking into other anatomical sites including the lung, peritoneal cavity, and the intestinal lamina propria (80–82). Consistent with the observation that IL-12 favors P- and E-selectin ligand synthesis more favorably than IL-4, Th1 CD4^+^ T cells traffic into the skin better than Th2 polarized cells (8, 83). As mentioned previously, PSGL-1 can function as both a P- and E-selectin ligand and its capacity to bind E-selectin regulates skin homing of Th1 CD4^+^ T cells (78). In contrast, CD43 becomes the dominant E-selectin ligand on Th17 CD4^+^ T cells (84). CD43 can also function as an E-selectin ligand in human T cells (85), but whether CD43 and PSGL-1 functioning as E-selectin ligands can be used to discriminate between Th17 and Th1 CD4^+^ T cells in either humans or mice has not been determined.

Several studies have suggested that the route of infection or vaccination will influence the trafficking potential of T cells following activation, a concept often referred to as “imprinting.” For example, CD8^+^ T cells activated in the draining lymph node following infection of the skin with Vaccinia virus begin synthesizing P- and E-selectin ligands, whereas the same antigen-specific CD8^+^ T cells activated in mesenteric lymph nodes when Vaccinia virus is delivered by intraperitoneal injection do not (86). Rather, this latter route of infection causes expression of the α_4_β_7_ integrin, which is generally considered to be critical for T cell homing to the gut. Dendritic cell-based vaccination strategies delivered either into the skin or intravenously have yielded similar findings (87). Mechanistically, production of the vitamin A metabolite, retinoic acid, by intestinal dendritic cells is able to actively suppress the synthesis of P- and E-selectin ligands during T cell activation, whereas dendritic cells from skin draining lymph nodes do not synthesize this metabolite (88–90). In CD4^+^ T cells, retinoic acid was shown to cause the promoter region of *Fut7* to remain methylated, thereby suppressing expression of this gene during T cell activation (91). Thus, understanding how different pathogens, routes of vaccination, or specific dendritic cell populations influence O-glycan synthesis and the trafficking potential of effector and memory T cell populations is critically important for the future development of rational vaccine designs.

In contrast to recently activated effector T cells, most memory CD8^+^ and CD4^+^ T cells do not actively synthesize core 2 O-glycans, which may limit their capacity to leave the circulation during homeostatic, non-inflammatory conditions. However, it has been appreciated for some time now that memory CD8^+^ T cells (and perhaps memory CD4^+^ T cells) acquire a promiscuous trafficking potential that allows them to traffic directly into areas of inflammation or tissue injury independent of antigen specificity or re-priming (92). We have recently shown that the memory CD8^+^ T cells that traffic into the lung or skin following inflammatory challenge or viral infection express core 2 O-glycans and that blocking P- and E-selectin prevents memory CD8^+^ T cells from trafficking into the inflamed skin (93). Memory CD8^+^ T cells exhibit an “open” epigenetic signature at the *Gcnt1* promoter and IL-15 (and potentially other cytokines that activate the STAT5 transcription factor) is sufficient to stimulate core 2 O-glycan synthesis, thereby allowing circulating memory CD8^+^ T cells to traffic into a site of infection without needing to be reactivated by professional antigen-presenting cells. This feature of memory CD8^+^ T cells contributes significantly to their ability to provide protective immunity against infections in non-lymphoid tissues. Whether core 2 O-glycan synthesis is regulated in a similar manner in memory CD4^+^ T cells has not been defined. Thus, these findings demonstrate that O-glycan synthesis can be activated and is highly dynamic in memory CD8^+^ T cells and that factors apart from antigen recognition control their tissue trafficking potential.

## Conclusion and Future Directions

The synthesis of complex O-glycans plays an indispensable role in regulating the trafficking of nearly every T cell type and subset *in vivo*, but the complexity of studying these forms of posttranslational modifications and lack of reliable and highly-specific reagents to monitor glycosylation status of cells or individual proteins has limited the progress of this field. Furthermore, the requirement for individual enzymes or proteins that function as selectin ligands to initiate extravasation and tissue homing have been studied most rigorously in neutrophils, not antigen-specific T cells. For example, it has not been determined if proteins such as CD44 and ESL-1 utilize primarily N- or O-glycans to function as E-selectin ligands in effector or memory T cell populations or if expression of these proteins impacts trafficking potentials. In addition, many of the studies identifying how O-glycan synthesis is stimulated on T cells have relied on *in vitro* activation strategies. Clearly, these reductionist approaches have strengthened our understanding of how diverse cytokines control the formation of P- and E-selectin ligands, but how these pathways are integrated *in vivo* during active infections or following vaccination is less understood. As we and others have shown, the capacity for memory T cells to rapidly traffic into a site of infection is critical for protective immunity and is highly dependent on *de novo* core 2 O-glycan synthesis. With regards to rational vaccine design, the process of imprinting memory T cell populations to home to a specific tissue microenvironment may be essential to generate functional memory T cells that are able to provide rapid and robust protective immunity. However, if and how diverse memory CD8^+^ and CD4^+^ T cell subsets (e.g., T_CM_ vs. T_EM_) or lineages (e.g., Th1 vs. Th17) regulate their O-glycan synthesis machinery *in vivo* remains mostly unexplored. Recently, a distinct lineage of tissue-resident memory (T_RM_) CD8^+^ T cells has been identified that is maintained for extended periods of time in non-lymphoid tissues. Not surprisingly, the formation of P- and E-selectin ligands is required to seed T_RM_ precursors in the skin during a viral infection (94). Interestingly, however, it was also reported that tissue-resident memory CD8^+^ T cells continue to synthesize E- and P-selectin ligands after they have established residency in the tissue microenvironment. The functional relevance for this is currently unknown, as it is not believed that this subset of memory CD8^+^ T cells ever re-enters the circulation. This suggests the possibility that the synthesis of O- and/or N-glycans by T_RM_ CD8^+^ T cells may be required for other biological functions in non-lymphoid tissues that have yet to be described. Finally, how O-glycan synthesis is regulated on antigen-specific T cells during either chronic infections or cancer has not been addressed. The latter, in particular, is of considerable interest clinically, as infiltration of CD8^+^ T cells into the tumor microenvironment is one of the most promising biomarkers associated with clinical response following immunotherapy. Thus, the therapeutic stimulation of core 2 O-glycans on tumor-specific T cells could be an attractive target for the rational design of combination therapies to enhance T cell trafficking and ultimately improve cancer immunotherapy techniques. Overall, the studies and findings described here highlight the importance of O-glycan synthesis in regulating the trafficking of essentially every type of T cell, and thus, deeper mechanistic understandings of this process could lead to advances in therapeutic interventions to either enhance or inhibit the activation and tissue infiltration of both protective (for pathogens and tumors) or pathogenic (for autoimmune or inflammatory disorders) antigen-specific T cells.

## Author Contributions

Both authors contributed equally to the writing of this review.

## Conflict of Interest Statement

The authors declare that the research was conducted in the absence of any commercial or financial relationships that could be construed as a potential conflict of interest.
